# 1,2,4-triazine derived binuclear lead(ii) complexes: synthesis, spectroscopic and structural investigations and application in organic light-emitting diodes (OLEDs)†

**DOI:** 10.1039/d4ra03383c

**Published:** 2024-07-12

**Authors:** Akbar Arkak, Moayad Hossaini Sadr, Mohammad Janghouri, Farzin Marandi, Daniel Fuhrmann

**Affiliations:** a Department of Chemistry, Faculty of Science, Azarbaijan Shahid Madani University Tabriz Iran; b Faculty of Industrial Technologies, Urmia University of Technology Band Road Urmia Iran; c Inorganic Chemistry Department, Faculty of Chemistry, Urmia University 57561-51818 Urmia Iran f.marandi@gmail.com; d Institut für Anorganische Chemie, Universität Leipzig Johannisallee 29 04103 Leipzig Germany

## Abstract

Two novel binuclear complexes of Pb(ii) were synthesized by reacting a 3-(2-pyridyl)-5-(4-methoxyphenyl)-1,2,4-triazine (PMPT) ligand with different anionic co-ligands (1: bromide, 2: acetate and isothiocyanate) in a 1 : 1 molar ratio of PMPT ligands to lead(ii) salts. The complexes, [Pb_2_(μ-PMPT)_2_Br_4_] (1) and [Pb_2_(μ-PMPT)_2_((μ-CH_3_COO)_2_(NCS)_2_] (2), were characterized using various physicochemical techniques such as CHN analysis, FT-IR spectroscopy, and ^1^H NMR spectroscopy. Additionally, their structures were determined using single-crystal X-ray diffraction. Based on the obtained structural parameters, complex 1 exhibited a PbN_3_Br_2_ environment, while complex 2 displayed a PbN_4_O_3_ environment, with holodirected and hemidirected coordination spheres, respectively. Within the crystal network of the complexes, there were interactions involving C–H⋯X (X: O, S, N) as well as π–π stacking. The Pb(ii) complexes were further investigated for their potential use as the emitting layer in organic light-emitting devices (OLEDs). The current–voltage and luminescence-voltage characteristics, as well as the electroluminescence (EL) properties of the complexes, were studied.

## Abstract 2

Synthesis, spectroscopic and structural characterization of two new binuclear lead(ii) complexes with PMPT ligand and further investigation for their potential use as the emitting layer in organic light-emitting devices (OLEDs).

## Graphical abstract

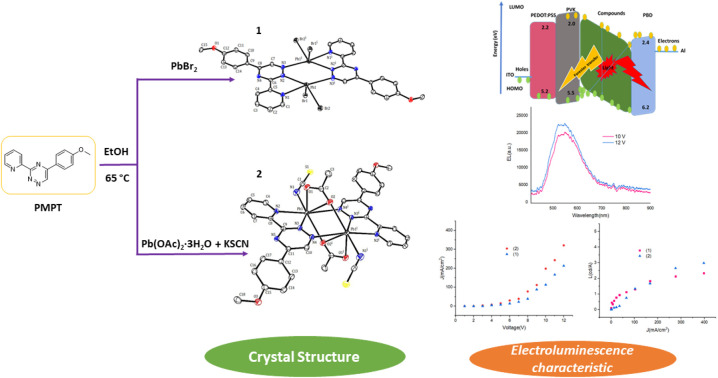

## 1. Introduction

π-Conjugated organic molecules possess highly versatile optoelectronic properties, such as light absorption and emission, that can be customized to a great extent. These molecules are already being used commercially in various areas, such as flexible electronics, optical displays, and logic circuits.^1–3^ Moreover, the application of organic compounds has witnessed remarkable interdisciplinary research in the fields of physics and materials science.^1–6^ Triazine derivatives are a well-known category of nitrogen-containing heterocyclic compounds. They serve as a fundamental component in a diverse array of applications, including optical bleaches, plastics, textiles, pharmaceuticals, rubber production, and surface-active agents.^3–5^ These derivatives have found extensive use as electron transport materials in organic light-emitting diodes (OLEDs), as thermally active delayed fluorescence emitters (TADF), and as donors in bulk heterojunction solar cells.^3–8^ An intriguing characteristic of triazine compound derivatives is their ability to form complexes with metal ions.^9^

The derivatives of 3-pyridyl-1,2,4-triazine have garnered attention due to their diverse coordination chemistry. For example, 3-(2-pyridyl)-5,6-diphenyl-1,2,4-triazine forms colored complexes with certain transition metal ions, offering potential applications in colorimetric analysis.^10^ Other derivatives, such as 2,4,6-tris[bis(pyridin-2-yl)amino]-1,3,5-triazine,^11^ 2,6-bis(tetramethylfuryl)-1,2,4-triazin-3-yl)pyridine,^12^ and 5,6-diphenyl-3-(2-pyridyl)-1,2,4-triazine,^13^ have been utilized in the construction of metal–organic frameworks (MOFs) with varying degrees of dimensionality.

Recently, complexes of lead(ii) with various ligands such as 3-(2-pyridyl)-5,6-diphenyl-1,2,4-triazine (pdpt),^14,15^ 3,5,6-tris(2-pyridyl)-1,2,4-triazine (tpt),^16,17^ 3-(2-pyridyl)-5,6-diphenyl-1,2,4-triazine-*p*,*p*′-disulfonate (pdpts),^18^ 3-(2-pyridyl)-5-phenyl-1,2,4-triazine (ppt),^19^ and 3-(2-pyridyl)-5,6-di(2-furyl)-1,2,4-triazine (pdft)^20,21^ were synthesized and characterized using X-ray crystallography. The influence of factors affecting the activity of the lead(ii) 6s^2^ lone pair in the coordination sphere was discussed. Building on this recent work, we present the synthesis, spectroscopic analysis, and structural study using experimental methods of two new binuclear lead(ii) complexes with the ligand 3-(2-pyridyl)-5-(4-methoxyphenyl)-1,2,4-triazine (PMPT): [Pb_2_(μ-PMPT)_2_I_4_] (1) and [Pb_2_(μ-PMPT)_2_(μ-CH_3_COO)_2_(NCS)_2_] (2). The optical and electrical properties of these complexes will be evaluated for potential application in organic light-emitting devices (OLEDs).

## 2. Experimental

### 2.1. Materials and measurements

All starting materials and solvents were purchased from commercial sources (Merck and Sigma-Aldrich) and used directly without any purification. The PXRD patterns were measured using a X-ray diffractometer (D500 S) utilizing Cu Kα (*λ* = 0.15418 nm) radiation source (30–40 kV and 40–50 mA) in the range of 2*θ* = 4–50°. The infrared spectra in the range 4000–400 cm^−1^ were recorded as KBr pellets with a FT-IR 8400-Shimadzu spectrometer. ^1^H NMR spectra were recorded using a Bruker DRX-250 Avance spectrometer at 250 MHz proton frequency; chemical shifts δ in ppm. ^1^H NMR spectra recorded in DMSO-*d*_6_ were referenced to the residual protonated signal of the solvent (2.47 ppm for DMSO-*d*_6_). Elemental analyses (C, H, N) were measured with a Thermo Finnigan Flash Elemental Analyzer 1112 EA. The melting points were determined with a Barnsted Electrothermal 9200 electrically heated apparatus. Thickness measurements were performed by DekTak 8000; Electrical and optical property of fabricated OLEDs were performed by USB2000 and HR4000 Ocean Optics. The current–voltage–luminance characteristics were checked by Keithley source meter 2400 model and optical meter Mastech-MS6612.

#### 2.1.1. Synthesis of 3-(2-pyridyl)-5-(4-methoxyphenyl)-1,2,4-triazine (PMPT) ligand

**Scheme 1:**
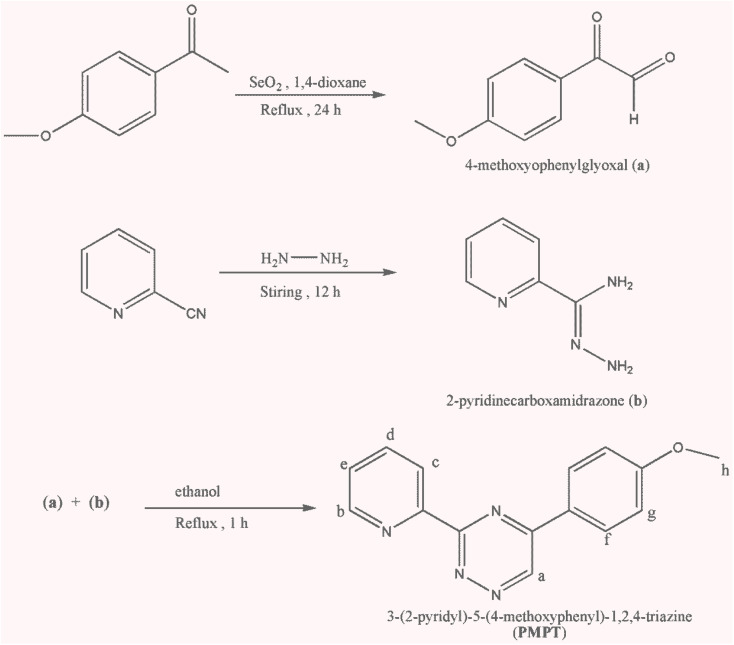
Synthesis steps of PMPT ligand.

The initial step involved synthesizing 4-methoxyphenylglyoxal (a) with certain modifications, following a procedure described in the literature.^22^ The starting material, 2-pyridinecarboxamidrazone (b), was prepared by mixing equimolar amounts of 2-cyano-pyridine and hydrazine monohydrate. A small quantity of ethanol was added until a clear solution formed. After allowing it to stand overnight at room temperature, the nearly colorless crystals of 2-pyridinecarboxamidrazone were filtered, washed with a small amount of ether, and air-dried. The preparation and purification of the 3-(2-pyridyl)-5-(4-methoxyphenyl)-1,2,4-triazine (PMPT) ligand from the reaction between compound (a) and compound (b) were carried out with some modifications, following procedures outlined in the literature^23^ (Scheme 1).

#### 2.1.2. Synthesis of [Pb_2_(μ-PMPT)_2_Br_4_] (1)

3-(2-pyridyl)-5-(4-methoxyphenyl)-1,2,4-triazine (PMPT) (0.27 g, 1.0 mmol) and PbBr_2_ (0.37 g, 1.0 mmol) were placed in the large arms of a branched tube. Ethanol was carefully added to fill both arms. The tube was then sealed and the ligand-containing arm was immersed in a bath at 65 °C while the other arm was maintained at ambient temperature.^24^ After a week, yellow crystals that were deposited in the cooler arm were filtered and dried in air. Yield: 0.39 g, 61%; m. p. 229 °C. Anal. Calcd for C_30_H_24_Br_4_N_8_O_2_Pb_2_ (1262.59): C, 28.51; H, 1.90; N, 8.87. Found: C, 28.27; H, 1.84; N, 9.09%. IR (KBr, cm^−1^): 3100 w (*ν*_CHar_), 2924 w (*ν*_CHaliphatic_), 1599 s and 1541 s (*ν*_C=N_), 1515 s (*ν*_C=C_)_ar_, 1266 s (*ν*_C_–_O_), 849 m and 770 m (*ν*_py_). ^1^H NMR (250 MHz, DMSO-*d*_6_, ppm, Hz): *δ* = 10.02)s, 1H_a_), 8.86)d, *J* = 2.5 Hz, 1H_b_), 8.51)d, *J* = 7.8 Hz, 1H_c_), 8.41) d, *J* = 8.5 Hz, 2H_f_), 8.05)t, *J* = 7.5 Hz, 1H_d_), 7.61 (t, *J* = 5.0 Hz, 1H_e_), 7.15 (d, *J* = 8.5 2H_g_), 3.85 (s, 3H_h_).

#### 2.1.3. Synthesis of [Pb_2_(μ-PMPT)_2_(μ-CH_3_COO)_2_(NCS)_2_] (2)

The procedure for synthesis of 2 was similar to 1 except that PbBr_2_ was replaced by Pb(CH_3_COO)_2_·3H_2_O (0.38 g, 1.0 mmol) and KSCN (0.10 g, 1.0 mmol). Orange crystals were formed after two days in the cooler arm and filtered. Yield: 0.30 g, 40%; m. p. 205 °C. Anal. Calcd for C_36_H_30_N_10_O_6_Pb_2_S_2_ (1177.2): C, 36.70; H, 2.55; N, 11.89. Found: C, 36.96; H, 2.77; N, 11.58%. IR (KBr, cm^−1^): 3059 w (*ν*_CHar_), 2950 w (*ν*_CHaliphatic_), 2049s (*ν*_isothiocyanate_), 1602 s and 1540 s (*ν*_C=N_), 1576 m (*ν*_asym_(COO)), 1515 s (*ν*_C=C_), 1412 m (*ν*_sym_(COO)), 1258 s (*ν*_C_–_O_), 842 m and 774 m (*ν*_py_). ^1^H NMR (250 MHz, DMSO-*d*_6_, ppm, Hz): *δ* = 10.04)s, 1H_a_), 8.83)d, *J* = 3.2 Hz, 1H_b_), 8.52)d, *J* = 7.8 Hz, 1H_c_), 8.42 (d, *J* = 8.8 Hz, 2H_f_), 8.05)t, *J* = 7.8 Hz, 1H_d_), 7.62 (t, *J* = 4.8 Hz, 1H_e_), 7.16 (d, *J* = 8.8 Hz, 2H_g_), 3.86 (s, 3H_h_), 1.69 (s, 3H_acetate_).

### 2.2. Crystal structure determination

Crystallographic data are given in Table 1. Single-crystal X-ray diffraction measurements were performed on a STOE IPDS 2T image plate diffractometer system equipped with a sealed Mo X-ray tube and a graphite monochromator crystal (*λ*(Mo-K_α_ = 0.71073 Å). Data reduction and numerical absorption correction were done with STOE X-AREA software.^25^ All structures were solved by direct methods using SHELXS-2018 and refined with SHELXL-2018 (ref. 26) using WinGX^27^ as a graphical frontend. All non-hydrogen atoms were refined with anisotropic thermal. Hydrogen atoms were included on idealized positions applying the riding model. Olex2 software^28^ and Diamond 3.2k^29^ were used for structural analyses and visualization. CCDC 2350383 and 2350384 contains the supplementary crystallographic data for this paper. These data can be obtained free of charge *via*https://www.ccdc.cam.ac.uk/structures/.

**Table 1:** Crystal data and structure refinement for complexes of 1 and 2

	1	2
Empirical formula	C_30_H_24_Br_4_N_8_O_2_Pb_2_	C_36_H_30_N_10_O_6_Pb_2_S_2_
Formula weight, g mol^−1^	1262.59	1177.2
Crystal size, mm^3^	0.24 × 0.15 × 0.06	0.13 × 0.12 × 0.10
Temperature, K	200(2)	200(2)
Crystal system	Triclinic	Triclinic
Space group	*P*1̄	*P*1̄

**Unit cell dimensions (Å, °)**		
*a*	7.7597(5)	9.7327(6)
*b*	9.1203(5)	10.2844(6)
*c*	12.5054(7)	10.5326(7)
*α*	102.924(4)	81.463(5)
*β*	97.329(4)	68.068(5)
*γ*	97.611(5)	86.765(5)
Volume, Å^3^	843.60(9)	967.1(2)
*Z*	1	1
Calculated density, g cm^−3^	2.485	2.021
Absorption coefficient, mm^−1^	14.78	8.88
*F*(000)	580	560
*θ* range for data collection (°)	2.68–27.00	2.68–28.00
*h*, *k*, *l* ranges	−9 ≤ *h* ≤ 9	−12 ≤ *h* ≤ 12
	−11 ≤ *k* ≤ 11	−13 ≤ *k* ≤ 13
	−15 ≤ *l* ≤ 15	−13 ≤ *l* ≤ 13
Reflections collected	6695	9247
Independent	3657	4574
*R*_int_	0.048	0.028
Data/ref. parameters	3657/209	4574/255
Goodness-of-fit on *F*^2^	1.011	0.984
Final *R* indexes [*I* >= 2*σ* (*I*)]	*R*_1_ = 0.0363	*R*_1_ = 0.0254
	*wR*_2_ = 0.0988	*wR*_2_ = 0.0556
Final *R* indexes [all data]	*R*_1_ = 0.0435	*R*_1_ = 0.0348
	*wR*_2_ = 0.1016	*wR*_2_ = 0.0572
Largest diff. peak/hole, e Å^−3^	1.88/−2.35	0.67/−1.57

### 2.3. Fabrication of OLEDs

**Fig. 1:**
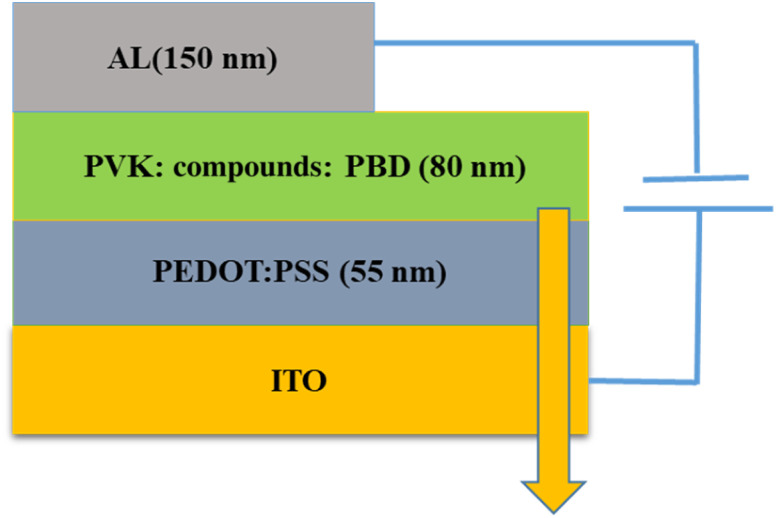
Schematic structures of the device.

ITO, were used as the conducting anodes. PEDOT: PSS as a hole injection layer was spin-coated on clean ITO substrate of 55 nm thickness and baked in an oven for 1 hour at 1200 °C. Afterward, PVK as a hole-transporting material and PBD as an electron-transporting material were doped with compound 1 and 2. PVK, PBD, and compound 1, 2 at ratio of 100 : 40 : 8 were blended in dimethylformamide (DMF) and then spin-coated and baked at 80 °C for 1 h. Finally, Al was evaporated by thermal evaporation method, respectively. Fig. 1 shows a schematic structure of the devices.

## 3. Results and discussion

### 3.1. Infrared spectra of complexes

The infrared (IR) spectra of compounds 1 and 2 were analyzed, and the selected frequencies are provided in Section 2. In the examined complexes, the stretching vibrations of the C=C, C=N, and N=N groups were observed at significantly lower values compared to the vibrations of *ν*(C=N) and *ν*(N=N) in the 3-(2-pyridyl)-5-(4-methoxyphenyl)-1,2,4-triazine (PMPT) ligand. This observation supports the coordination of the triazine and pyridyl rings to the metal ion.^30^ The weak band observed at 2950–3000 cm^−1^ is attributed to the methoxy group of the PMPT ligand and the acetate *ν*(CH) mode in compound 2. In 2, the bands around 1576 and 1412 cm^−1^ correspond to the modes *ν*_asym_(COO) and *ν*_sym_(COO), respectively, indicating the presence of the acetate ligand in the molecule. Previous attempts have been made to correlate the positions of these modes or the frequency difference Δ*ν* (*ν*_asym_ − *ν*_sym_) with the bonding type.^31^ The Δ*ν* value of acetate in the lead(ii) complex is 164 cm^−1^, which aligns with the expected bidentate and bridging coordination of acetate. Additionally, the bands at a frequency of 2049 cm^−1^ in the IR spectrum of 2 provide evidence of N-coordination between the terminal isothiocyanate anions and the lead center, as supported by the crystal structure^32^ (Fig. S1 and S2†).

#### 3.1.1. ^1^H NMR spectroscopy of complexes

The ^1^H NMR spectra of all compounds were obtained in DMSO-*d*_6_ and clearly show the presence of the PMPT ligand. In the spectrums, peaks ranging from 7.65 to 10.13 ppm confirm the aromatic nature of the ligand. A single peak at the lowest magnetic field (10 ppm) corresponds to the hydrogen atom in the triazine ring (H^a^). The signal at 8.8 ppm can be attributed to the hydrogen near the pyridine nitrogen atom (H^b^). The peaks representing the other hydrogen atoms in the pyridine ring appear between 7.47 and 8.90 ppm, manifesting as two doublets of doublets (H^d^ and H^e^) and one doublet (H^c^). The hydrogen atoms in the phenyl ring are observed as two doublets at 7.15 and 8.4 ppm (H^f^ and H^g^). A singlet peak at 3.8 ppm corresponds to the three hydrogen atoms in the methoxy group (H^h^). In the spectrum of compound 2, in addition to the mentioned peaks, a singlet peak at the highest magnetic field (1.69 ppm) is observed, which represents the hydrogen atoms of the acetate anion (Fig. S3 and S4†).

### 3.2. Crystal structure description

The crystalline phase purities for 1 and 2 were confirmed by the PXRD patterns. Fig. S5† shows that the powder patterns of the two complexes match quite well with those simulated from single-crystal X-ray data, indicating the bulk purities of the complexes.

**Scheme 2:**
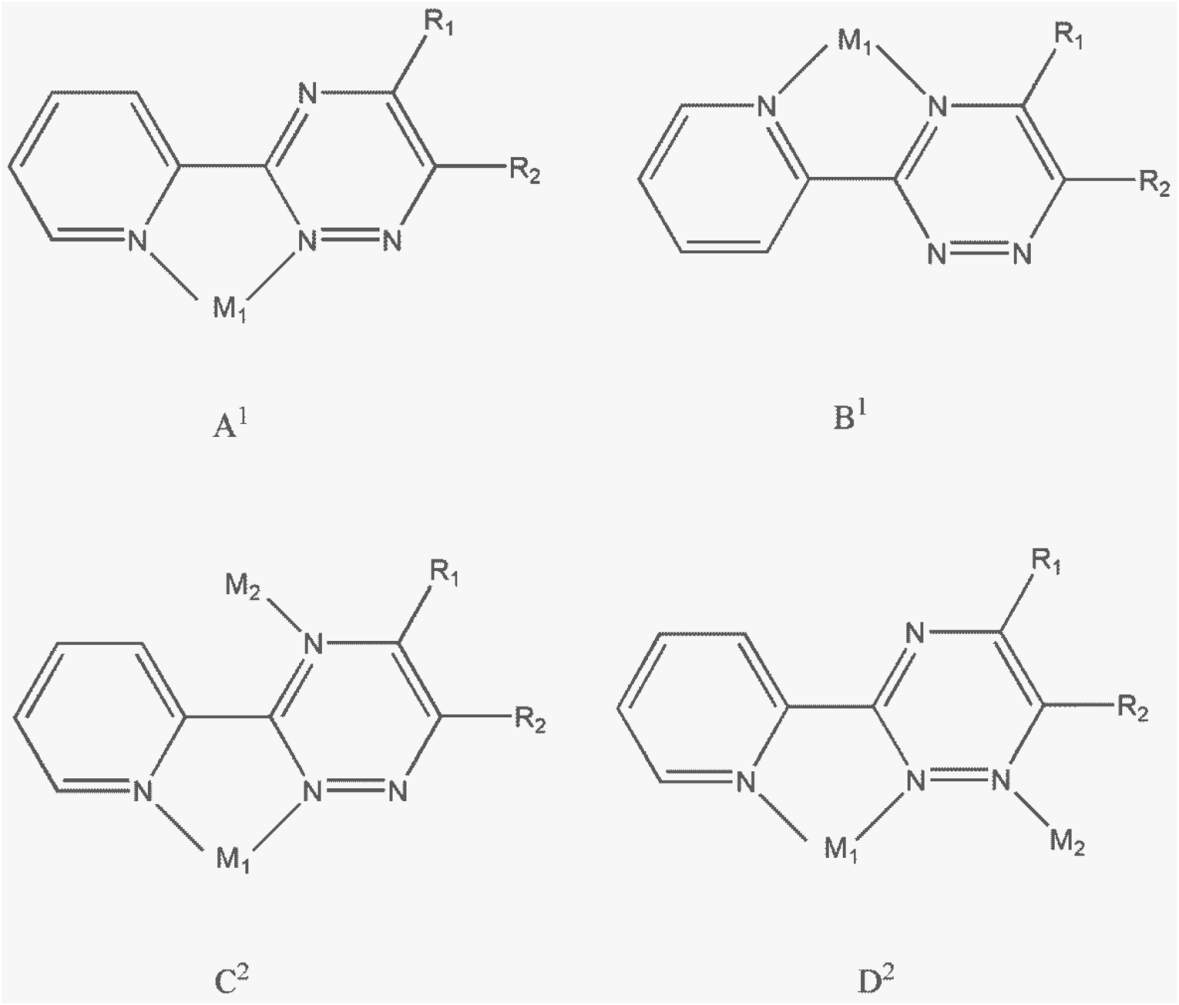
Possible coordination modes of derivatives of 3-(2-pyridyl)-1,2,4-triazine (PTZ) ligands to form mononuclear (A^1^, B^1^) or binuclear (C^2^, D^2^) metal complexes.

Scheme 2 illustrates the various possible coordination modes of derivatives of 3-(2-pyridyl)-1,2,4-triazine (PTZ) ligands. The PTZ ligands can form mononuclear lead complexes through a bidentate coordination site similar to 2,2′-bipyridine (referred as form A^1^). These mononuclear complexes are commonly described in the literature as derivatives involving PTZ ligands.^14,15,18–21^ By combining bidentate and monodentate coordination sites provided by the PTZ ligands, it is possible to obtain dinuclear complexes (referred to as forms C^2^ and D^2^). Only one binuclear complex of silver(i) in coordination form D^2^ has been reported.^33^ However, there are no examples in the literature where PTZ ligands coordinate to lead atoms in coordination forms B^1^, C^2^, and D^2^. In this study, we have synthesized two binuclear complexes where the PMPT ligand coordinates to the lead atom in coordination form D^2^.

**Fig. 2:**
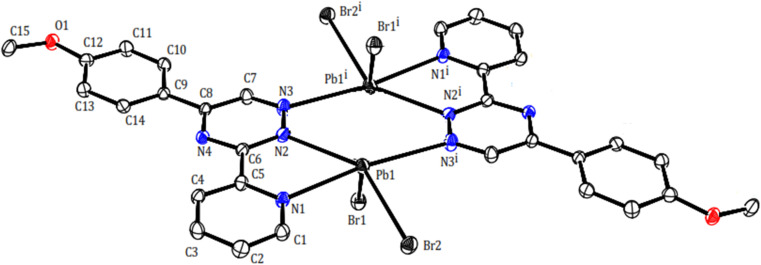
Molecular structure of compound 1 with atom numbering scheme.

**Fig. 3:**
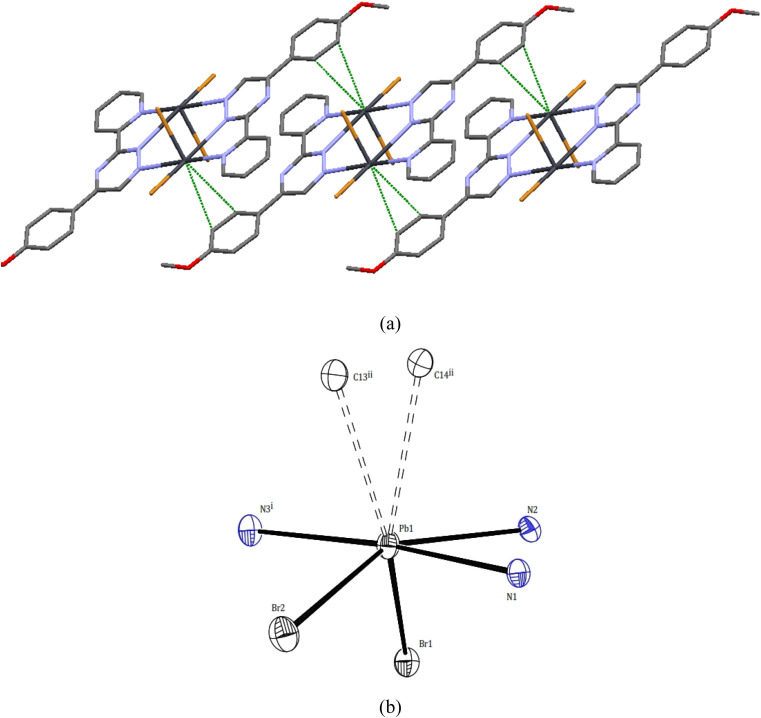
(a) Pb⋯C tetral bonding and π–π stacking (b) lead atoms geometries in 1.

Two novel lead(ii) complexes with the newly developed triazine ligand, 3-(2-pyridyl)-5-(4-methoxyphenyl)-1,2,4-triazine (PMPT), and varied anions were synthesized and analyzed by their influences on the coordination chemistry of lead(ii). X-ray single-crystal structure analyses indicate that [Pb_2_(μ-PMPT)_2_Br_4_] (1) crystallizes in the triclinic space group *P*1̄ (Table 1), with one formula unit per unit cell. A molecular view and selected bond parameters of 1 are presented in Fig. 2 and Table 2. Considering the PMPT ligand as a bidentate and monodentate N-donor ligand, along with two bromide anions, the compound [Pb_2_(μ-PMPT)_2_Br_4_] suggests the presence of a five-coordinate Pb atom. This coordination number is relatively low for Pb(ii) in an N and Br-donor environment. The structure solution reveals the Pb atoms occurring in pairs that are approximately 3.900(2) Å apart. These pairs are formed through N-bridging in a centrosymmetric dimer unit consisting of PbN_3_Br_2_ entities. The structure of this “dimer” unit is quite remarkable, as depicted in Fig. 3, displaying a highly “hemidirected” coordination sphere. This observation suggests that this system might serve as an example of a stereochemically active lone pair. Within a “hemidirected” coordination sphere, the length of the Pb–X bonds varies and is generally greater than 0.4 Å. This variation is influenced by their proximity to a stereochemically active lone pair or their distance from it.^34^ However, in the case of compound 1, there is an approximate difference of 0.2 Å in the lengths of the five Pb–N and Pb–Br bonds. Notably, these longer bond lengths are indicative of a “holoidirected” coordination sphere.

**Table 2:** Selected bond lengths (Å) and angles (°) for complexes of 1 and 2

1					
Pb1–N1	2.624(5)	Pb1–N2	2.743(5)	Pb1–N3^i^	2.818(6)
Pb1–Br1	2.792(8)	Pb1–Br2	2.828(7)	Pb1⋯C14^ii^	3.465(6)
Pb1⋯C13^ii^	3.538(6)	N1–Pb1–N2	60.84(2)	N1–Pb1–Br1	97.69(1)
N2–Pb1–Br1	74.14(1)	N1–Pb1–Br2	88.72(1)	N2–Pb1–Br2	145.48(1)
Br1–Pb1–Br2	96.13(2)	N1–Pb1–N3^i^	172.87(1)	Br2–Pb1–N3^i^	84.33(1)
i 1 − *x*, 2 − *y*, 1 − *z*; ii 1 − *x*, 1 − *y*, 1 − *z*					

2					
Pb1–O1	2.402(4)	Pb1–O2	2.601(4)	Pb1–N1	2.624(5)
Pb1–N2	2.629(3)	Pb1–O2^i^	2.746(3)	Pb1–N3	2.762(4)
Pb1–N4^i^	2.951(3)	Pb1⋯S1^ii^	3.441(2)	O1–Pb1–O2	51.38(1)
O1–Pb1–N1	77.54(1)	O2–Pb1–N1	115.90(1)	O1–Pb1–N2	76.37(1)
O2–Pb1–N2	113.00(1)	N1–Pb1–N2	83.65(1)	O1–Pb1–O2^i^	131.13(1)
O2–Pb1–O2^i^	82.27(1)	N1–Pb1–O2^i^	146.72(1)	N1–Pb1–N4^i^	89.70(1)
N2–Pb1–O2^i^	116.12(1)	N2–Pb1–N3	61.26(1)	N3–Pb1–N4^i^	123.59(1)
i 1 − *x,* 2 − *y,* 1 − *z*; ii 2 − *x*, 2 − *y*, 1 − *z*					

Upon further examination, it becomes clear that the interpretation of the crystal structure mentioned above is an oversimplification. The chains of Pb atom pairs, aligned parallel to the crystallographic *c*-axis, arise from close “intermolecular” contacts between such dimeric units. This arrangement is illustrated for a single adjacent pair in Fig. 3. While the parallel alignment of certain phenyl groups may initially suggest the presence of π-stacking interactions throughout the lattice, it is actually a consequence of two-hapto interactions occurring between the Pb atoms and phenyl groups originating from separate dimers.

A search was made generally for Pb⋯C approaches and it appears that Pb atoms in compound 1 may also be involved in *η*^2^ interaction with the phenyl groups of another dimer. Thus, the Pb atoms are linked to two carbon atoms of phenyl groups, with distances Pb1⋯C14^ii^ and Pb1⋯C13^ii^ of 3.465(6) and 3.538(6) Å, respectively. Hence, the Pb^II^ coordination sphere is completed and rather than a PbN_3_Br_2_ coordination sphere, the complex can be considered to contain a dihapto interactions (PbC_2_N_3_Br_2_) center with an irregular seven coordination number but ‘‘holodirected” coordination sphere (Fig. 3a and b). The reported Pb⋯C separations range is 3.083–4.05 Å in species [Pb(*o*-xylene)_2_(Cl_2_AlCl_2_)_2_], [Pb(*η*^6^-C_6_H_6_)(Cl_2_AlCl_2_)_2_]C_6_H_6_ (ref. 35) [Pb_2_{SeC_6_H_2_(CF_3_)_3_}_4_(toluene)_2_]^36^ and [Pb_2_(DBM)_4_].^37^ Thus, Pb⋯C interactions in compound 1 appear to be yet another factor which can make varying contributions to the stability of complexes of this metal ion.

Within the crystal structure of complex 1, adjacent complex molecules are connected through robust intermolecular hydrogen bond interactions. The primary intermolecular interactions observed in compound 1 include C–H⋯O and C–H⋯Br hydrogen bonds, as well as π⋯π stacking interactions between the molecules. These interactions play a crucial role in determining the overall arrangement of the crystal packing and contribute to the stabilization of the crystal structure in a two-dimensional supramolecular manner. For further details, refer to Fig. S6† and Table 3.

**Table 3:** Hydrogen bond and intermolecular interactions (Å and °) in complexes of 1 and 2

D–H···A	*d*(D–H)	*d*(H⋯A)	<(DHA)	*d*(D⋯A)	Symmetry code on A atom
1					
C2–H2⋯O1	0.950	2.524	135.42	3.269(8)	−1 + *x*, *y*, −1 + *z*
C4–H4⋯Br1	0.950	2.848	148.42	3.691(7)	*x*, −1 + *y*, *z*
π–π (slipped face to face) between the phenyl and pyridyl rings				3.370(2)	2 − *x*, 1 − *y*, 1 − *z*

2					
C7–H7⋯O1	0.949	2.597	154.28	3.477(1)	1 − *x*, 1 − *y*, 1 − *z*
C17–H17⋯O1	0.980	2.564	158.10	3.464(3)	1 − *x*, 1 − *y*, 1 − *z*
π–π (slipped face to face) between the triazine and pyridyl rings				3.359(2)	1 − *x*, 1 − *y*, 1 − *z*

**Fig. 4:**
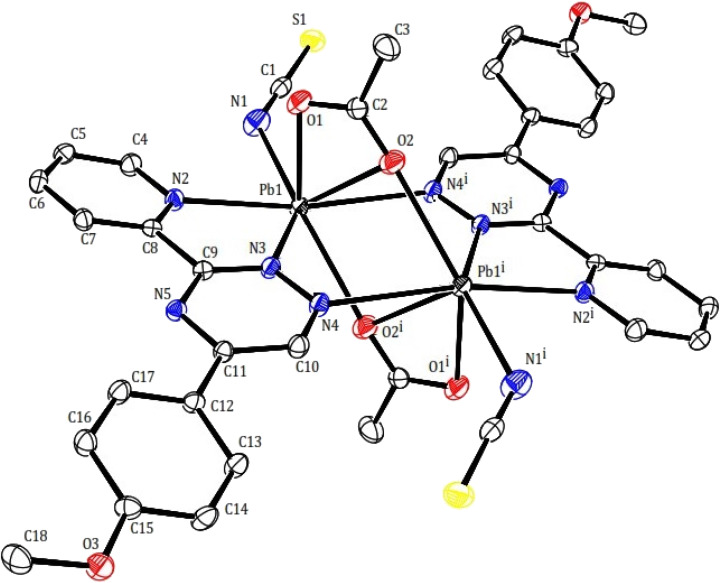
Molecular structure of compound 2 with atom numbering scheme.

In compound 2, the structure may be considered as a coordination polymer of lead(ii) consisting of dimeric units with a building block of [Pb_2_(μ-PMPT)_2_(μ-CH_3_COO)_2_(NCS)_2_] (2). Similarly to compound 1, Two PMPT ligand doubly bridge two lead(ii) ions *via* the N atoms (nitrogen of pyridine and triazine as chelating and another nitrogen of triazine as monodentate to another lead atom). The dimeric units are further linked across a center of inversion by two acetate anions, resulting are shown in Fig. 4. The Pb⋯Pb distances within the 2 moieties, those bridged by the acetate anions are 4.027(3) Å. Within the dimer unit, the carboxylate moiety of each acetate ligand acts as both bidentate, and bridging group (totally tridentate) in a μ-1,3 mode: both oxygen atoms of the carboxylate group coordinate to a lead(ii) ions yielding the Pb_2_O_2_ core. Isothiocyanate anions as acts monodentate and as terminal N donor. Thus per lead atoms in 2 is seven-coordinated (PbN_4_O_3_) by two nitrogen of two PMPT ligands, nitrogen of isothiocyanate anion and three oxygen of two acetates with the Pb–O distances of 2.402(4), 2.601(4), 2.746(3) Å and Pb–N distances of 2.624(5), 2.629(3), 2.762(4) 2.762(4) and 2.951(3) Å (Table 2). This arrangement created a gap or hole in coordination geometry around the metal ions (presence of gap is clear), occupied possibly by a “stereoactive” lone pair of electrons on lead(ii), and the coordination sphere is hemidirected. The bond length difference more than 0.4 Å in the coordination sphere and the observed shorting of Pb–O bonds on the side of Pb^2+^ ion opposite to the putative lone pair supports the presence of the lone pair electrons.^34^

**Fig. 5:**
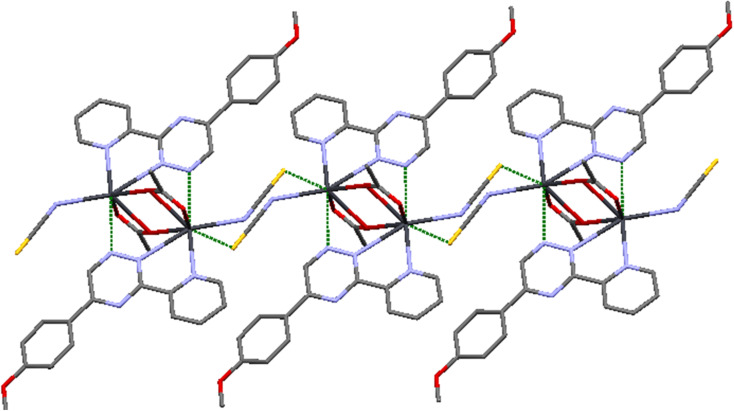
Pb1⋯S tetral bonding in 2.

This particular environment provides a suitable space for forming bonds with other atoms. To explore potential donor centers, it is necessary to extend the bonding range. Within a limit of 3.5 Å (which is smaller than the van der Waals radius), there are Pb⋯S(thiocyanate) tetrel bonds observed in the crystallographic [010] direction. These bonds have distances of 3.441(2) Å, connecting the dimers and forming a polymeric chain. These distances fall within the sum of the van der Waals radii^38^ of the corresponding atoms. The Pb⋯S tetral distance in compound 2 is similar to that reported for lead(ii) complexes with thiocyanate (Fig. 5).^39^ Additionally, the Pb⋯Pb distances within the [Pb_2_(μ-SCN)_2_]*n* moieties measure 6.716 Å.

In compound 2, various types of interactions, including intermolecular, intramolecular, and π–π stacking interactions, contribute to the arrangement of the complex in the crystal lattice (refer to Table 3). To investigate the presence of weak directional intermolecular interactions in 2, Mercury programs were utilized. The analysis revealed the existence of C–H⋯O^40^ interactions and π–π stacking. The packing diagram of 2 demonstrates a two-dimensional self-assembled structure formed through slipped face-to-face π–π stacking. Notably, the distance between the triazine and pyridyl rings measures 3.359(2) Å, which is considerably shorter than the typical distance observed in normal π–π stacking.^41^ Furthermore, the two-dimensional supramolecular networks in 2 are constructed through C–H⋯O interactions. The distances for these interactions are 2.597 and 2.564 Å, significantly shorter than the van der Waals distance of 2.77 Å for H⋯O interactions.^38^ Refer to Fig. 7S† for visual representation.

### 3.3. Optical characteristics

**Fig. 6:**
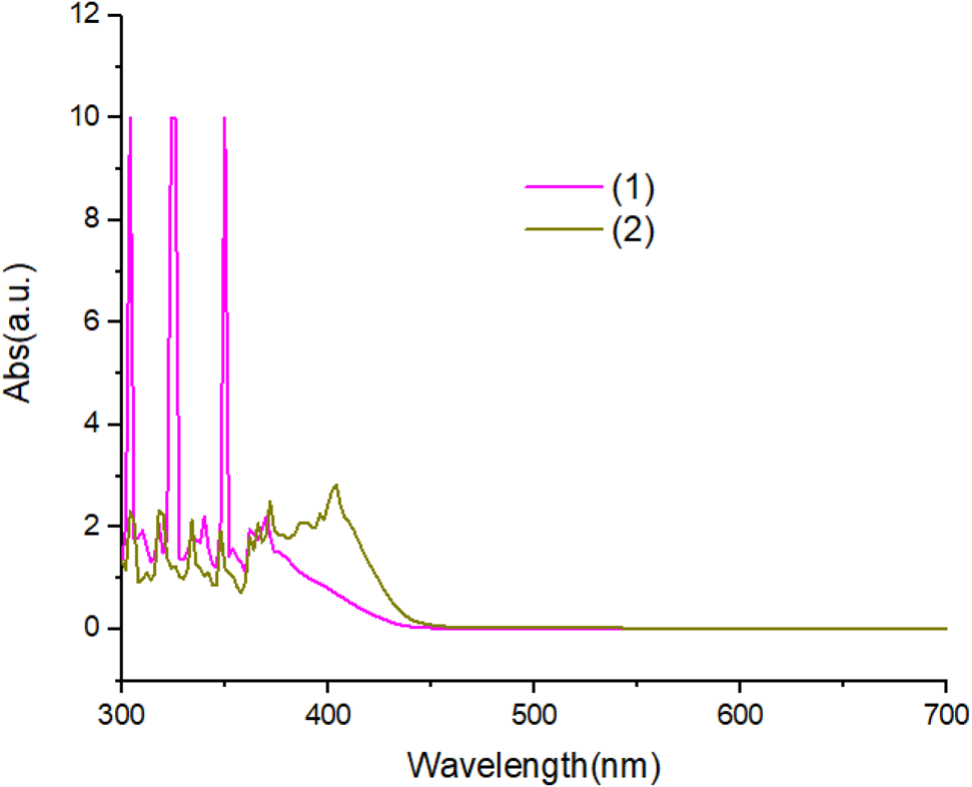
The UV-Vis absorption spectra of compounds.

The UV-Vis absorption spectra of complexes in ethanol solution are shown in Fig. 6. Compound 1 exhibit two absorption peaks at 380 nm and 360 nm. The UV-Vis spectrum of compound 2 is red shifted with respected to compound 1. All peaks can be attributed to the π–π* transitions of the aromatic ligand.

**Fig. 7:**
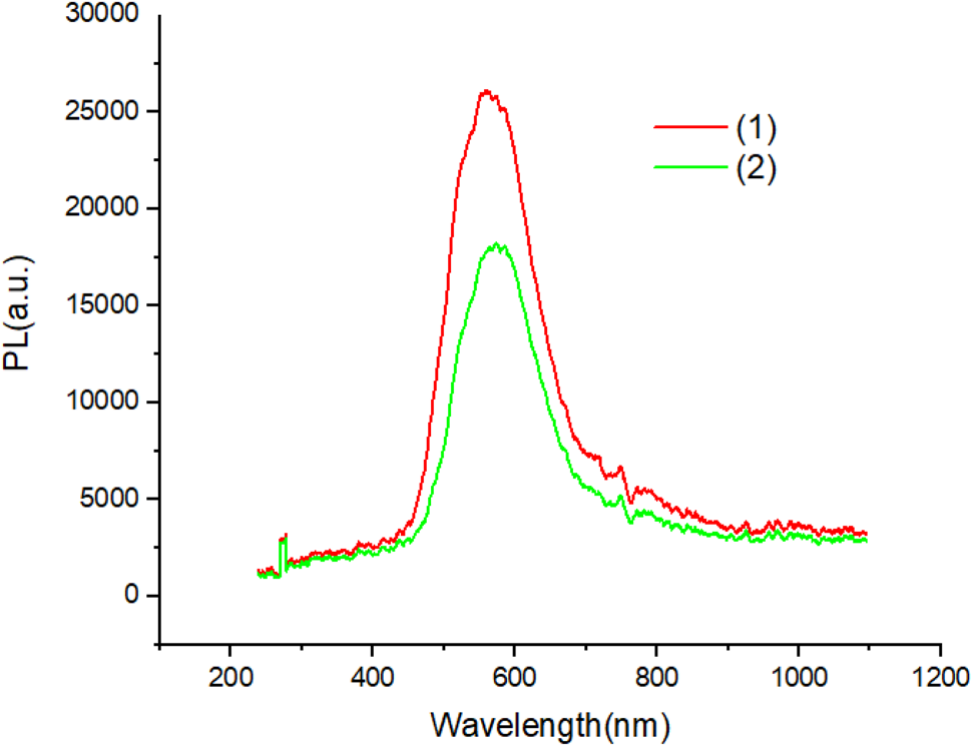
The PL of solution of compounds.

**Fig. 8:**
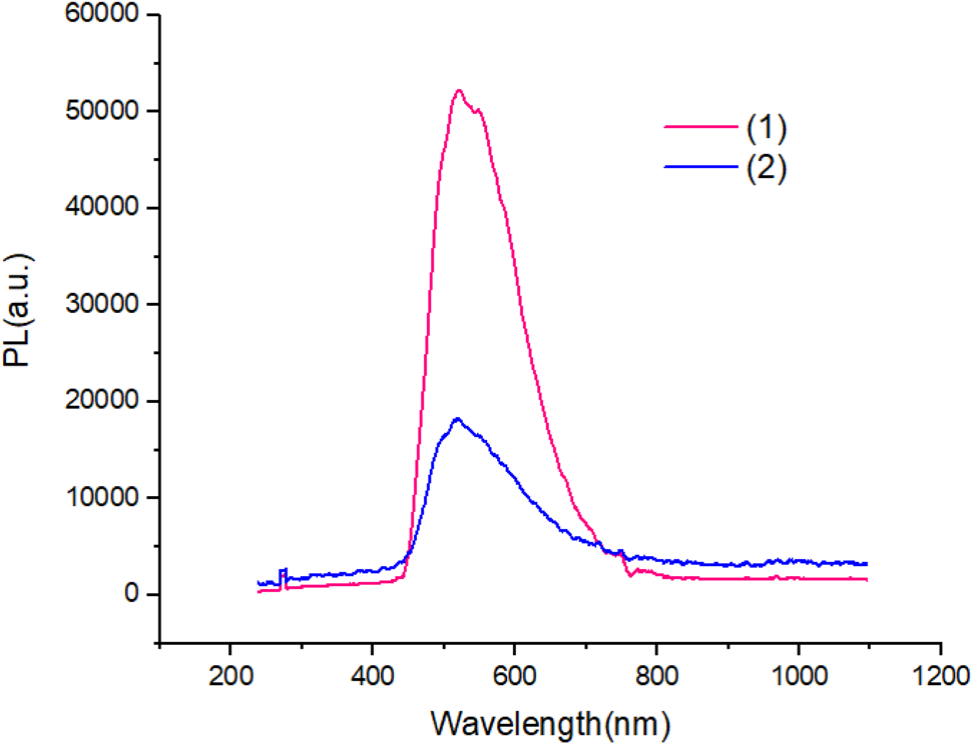
The PL of solid state of compounds.

Fig. 7 and 8 shows the characteristics of the PL solution and solid state of compounds in the solution state by exciting with a wavelength of 405 nm, the PL spectra of compounds shows peak emission spectrum centered at 596 nm, but in the solid state, the PL spectra of compounds blue shifted about 65 nm with respect to solution.

The PL quantum yields of the compounds were achievement using the equation established by Parker and Rees.^42,43^ The highest PL quantum yield for compounds 1 and 2 are measured as 0.67, and 0.38, respectively. The Förster radius energy transfer rate is commonly employed to showcase the power efficiency in OLED technology. The Förster radius is defined as:
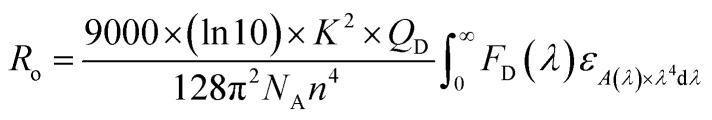
Here, *k*^2^, *N*_A_, *Q*_D_, *n*, *F*_D_(*λ*) and *ε*_A_(*λ*) represent the orientation factor, Avogadro's number, donor quantum yield, refractive index, fluorescence intensity, and extinction coefficient. The Förster radius values for compounds 1 and 2 are measured as 3.34 nm and 4.67 nm, respectively. Table 4 shows the optical property of compounds.

**Table 4:** The optical properties of compounds

Complex	*k* ^2^	NA	QD	*n*	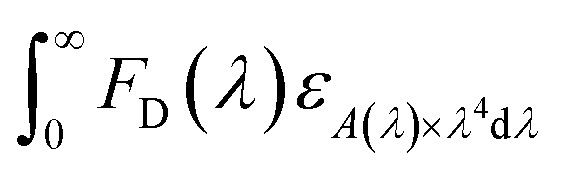
1	2/3	6.02 × 10^23^	0.67	1.73	2.5 × 10^−32^
2	2/3	6.02 × 10^23^	0.38	1.68	2.2 × 10^−34^

**Fig. 9:**
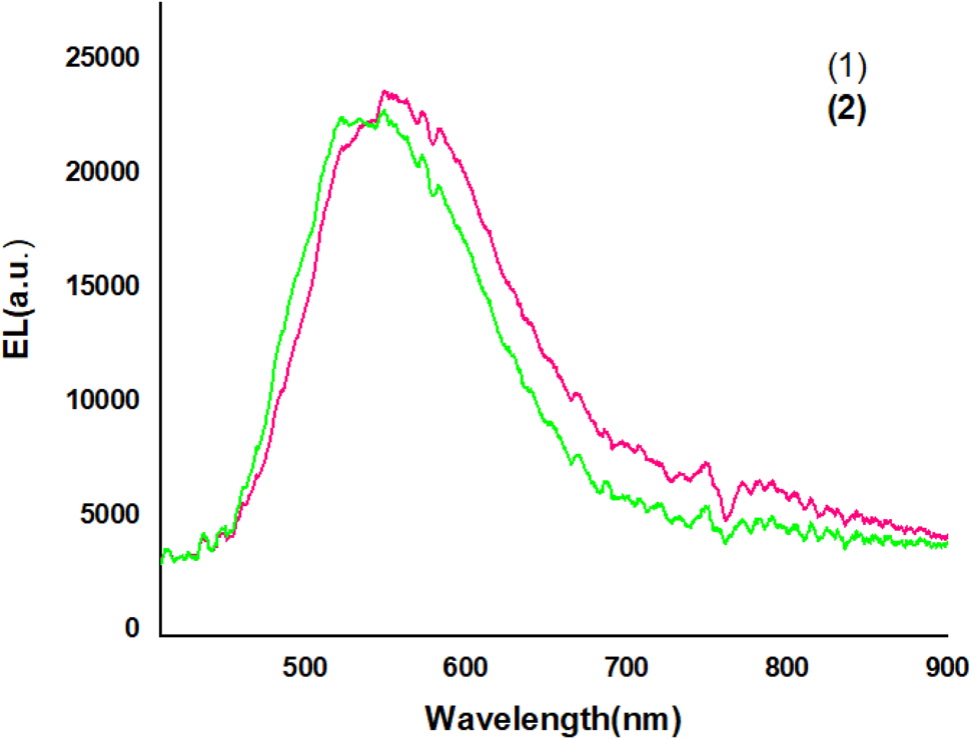
The EL spectra of devices.

Fig. 9 shows The EL spectra of OLED devices. The EL of the compound 1 and 2-based devices showed a band in the green and yellow regions, respectively.

**Fig. 10:**
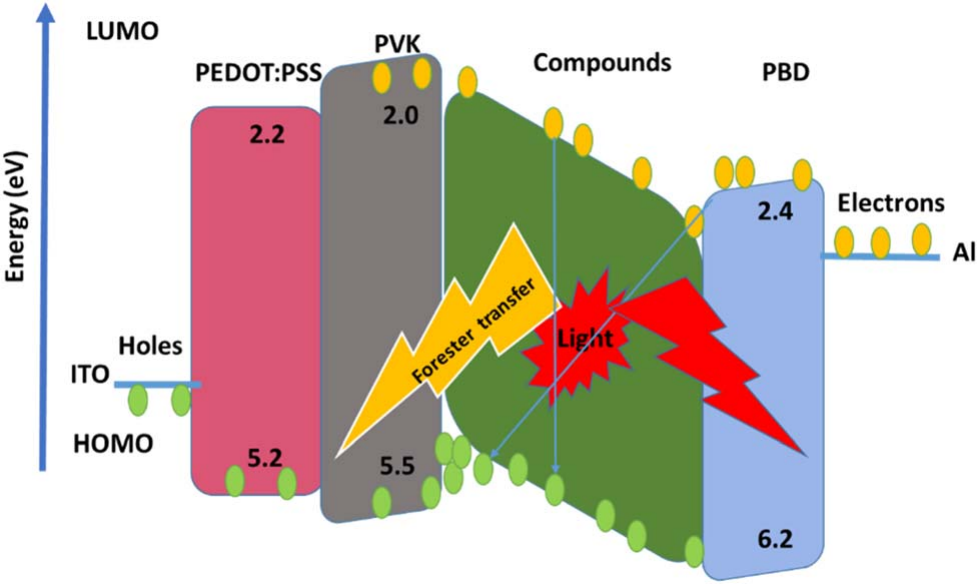
Schematic energy levels.

The EL spectra of the OLEDs depended on the PbN_3_Br_2_, 1 and PbN_4_O_3_, 2 of the environment of Pb complexes. The emission wavelength shifted from the green color to the yellow color when the functional group varied from PbN_3_Br_2_ to PbN_4_O_3_. (see the Fig. 10).

**Fig. 11:**
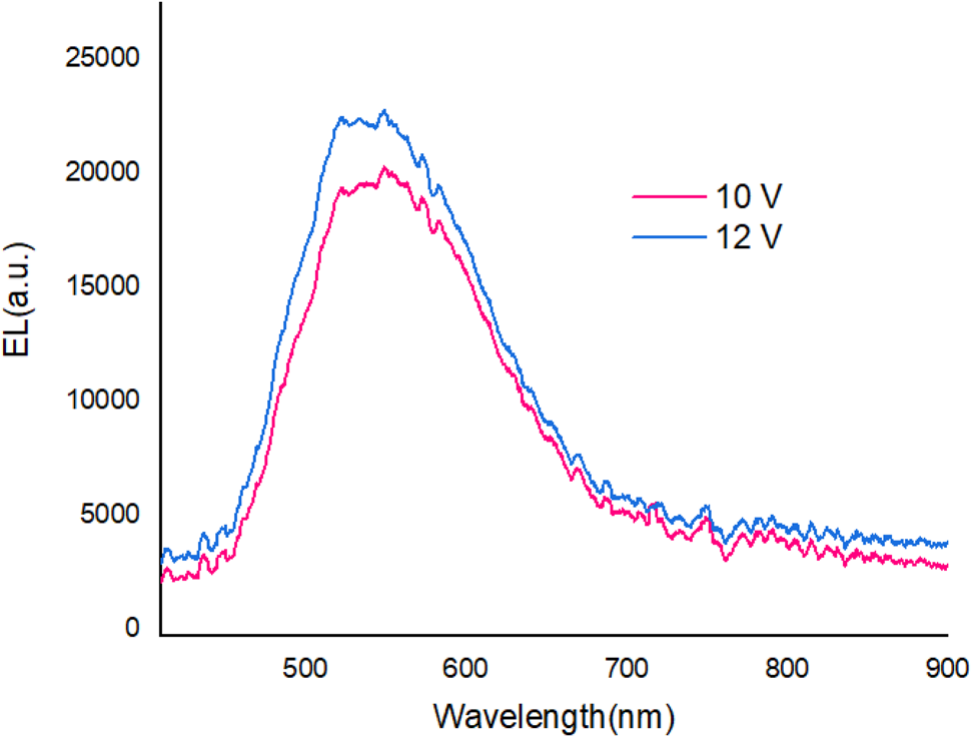
The EL spectrum at various voltages.

With applying of voltage, electrons (e) and holes (h) injected in the PEDOT:PSS and PBD layer, Finally e/h recombines at the compound molecules. Also, the EL intensity at 560 nm (Fig. 11) dependent on the applying voltage and with increasing voltage the EL intensity increases.

**Fig. 12:**
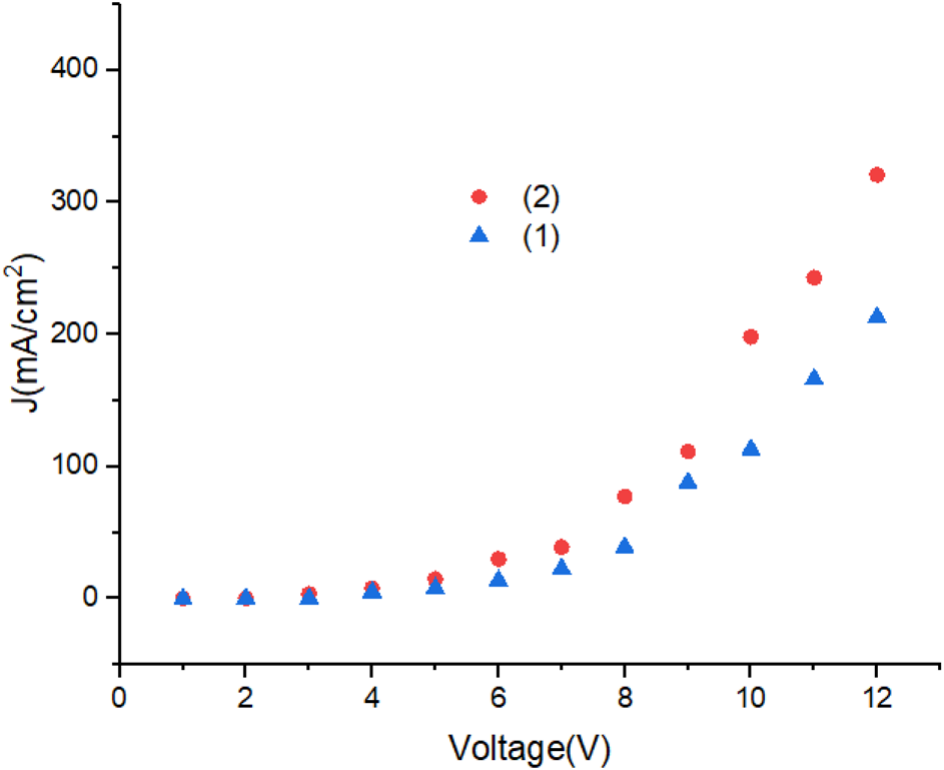
The current density–voltage characteristic of device.

Fig. 12 shows the current density of the devices. Turn on voltage of the OLEDs is lower than 4.5 V. With increasing of voltage the current density increases. The J–V characteristics change from ohmic region to the space charge limited current (SCLC) region, respectively. A significant improvement in the electrical conductivity in OLEDs can be achieved by modification of the molecular structure.^44–47^

**Fig. 13:**
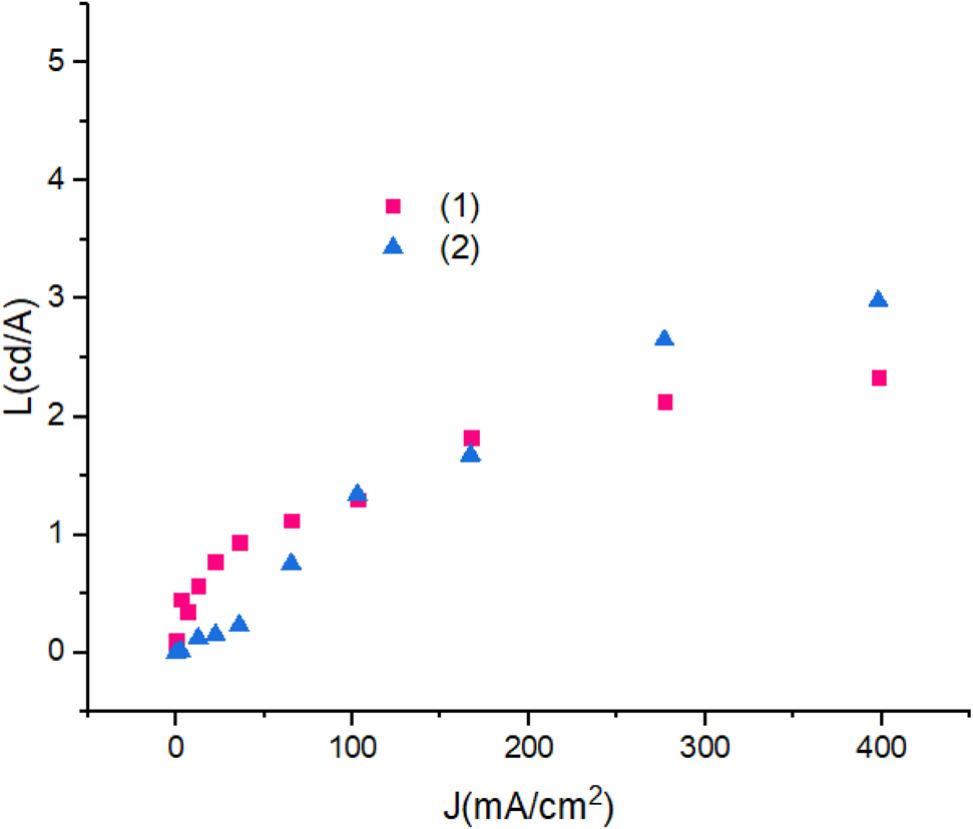
Luminescence efficiency–current density characteristic of devices.

Fig. 13 shows luminescence efficiency – current density of devices. The differences between the luminescence are ascribed to the role of the PbN_3_Br_2_ to PbN_4_O_3_ in compounds. Also, with the increase in applied current density the luminescence remains reliably stable.

## 4. Conclusion

In conclusion, we report the synthesis and physicochemical structural characterization of two new binuclear Pb(ii) complexes with PMPT ligand and auxiliary anionic co-ligands: bromide in 1, acetate and isothiocyanate in 2. In all complexes, per PMPT ligand coordinate two lead atoms by three nitrogen atoms (new coordination mode in lead complexes). Based on the obtained structural parameters, complex 1 exhibited a PbC_2_N_3_Br_2_ environment, while complex 2 displayed a PbN_4_O_3_S environment, with holodirected and hemidirected coordination spheres, respectively. It seems that anions play an important role in the activity of lead(ii) coordination sphere by creating various intermolecular interactions. Upon closer examination, it becomes evident that tetrel bonding, although not adequately recognized until now, plays a significant role in the study of lead's solid-state chemistry. This newfound understanding has the potential to greatly aid in the manipulation and design of supramolecular architectures and organometallic frameworks that rely on lead coordination complexes. The prepared complexes show high luminescence efficiency at room temperature and have good stability, which make it suitable for the fabrication of OLEDs. It should be noted that auxiliary ligands are a factor for changing the optical properties of compounds. The results of this work show that these compounds can be used as a precursor in the manufacture of optical devices.

## Data availability

CCDC 2350383 and 2350384 contain the supplementary crystallographic data for this paper. These data can be obtained free of charge *via*https://www.ccdc.cam.ac.uk/structures/.

## Conflicts of interest

There are no conflicts to declare.

## Supplementary Material

RA-014-D4RA03383C-s001

RA-014-D4RA03383C-s002
