# Supplementary material for: 1,2,4-triazine derived binuclear lead(ii) complexes: synthesis, spectroscopic and structural investigations and application in organic light-emitting diodes (OLEDs)
Source: RSC Adv. 2024 Jul 12;14(30):22006–16. doi: 10.1039/d4ra03383c (PMC11240088; doi:10.1039/d4ra03383c)
Supplement: RA-014-D4RA03383C-s001 [file RA-014-D4RA03383C-s001.pdf]

Electronic Supplementary Material (ESI) for RSC Advances.
This journal is © The Royal Society of Chemistry 2024

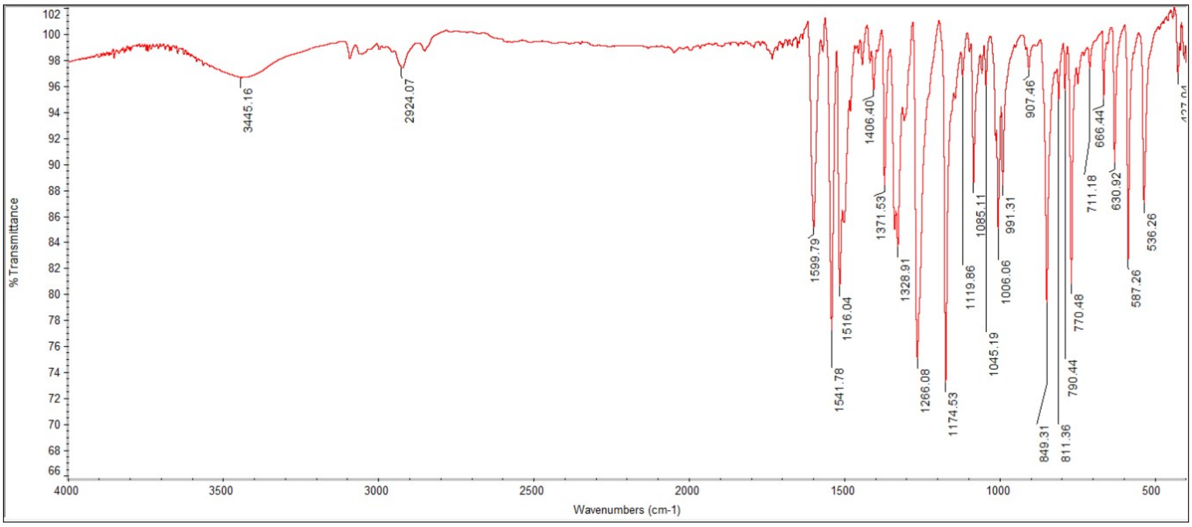

Figure S1: FT-IR spectrum of **1**.

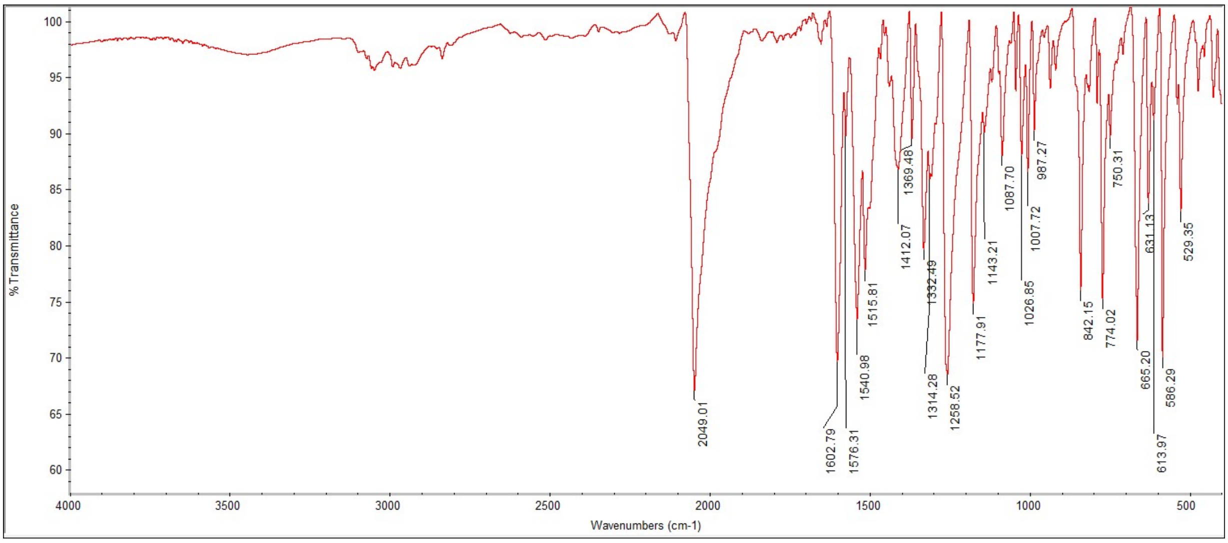

Figure S2: FT-IR spectrum of **2**.

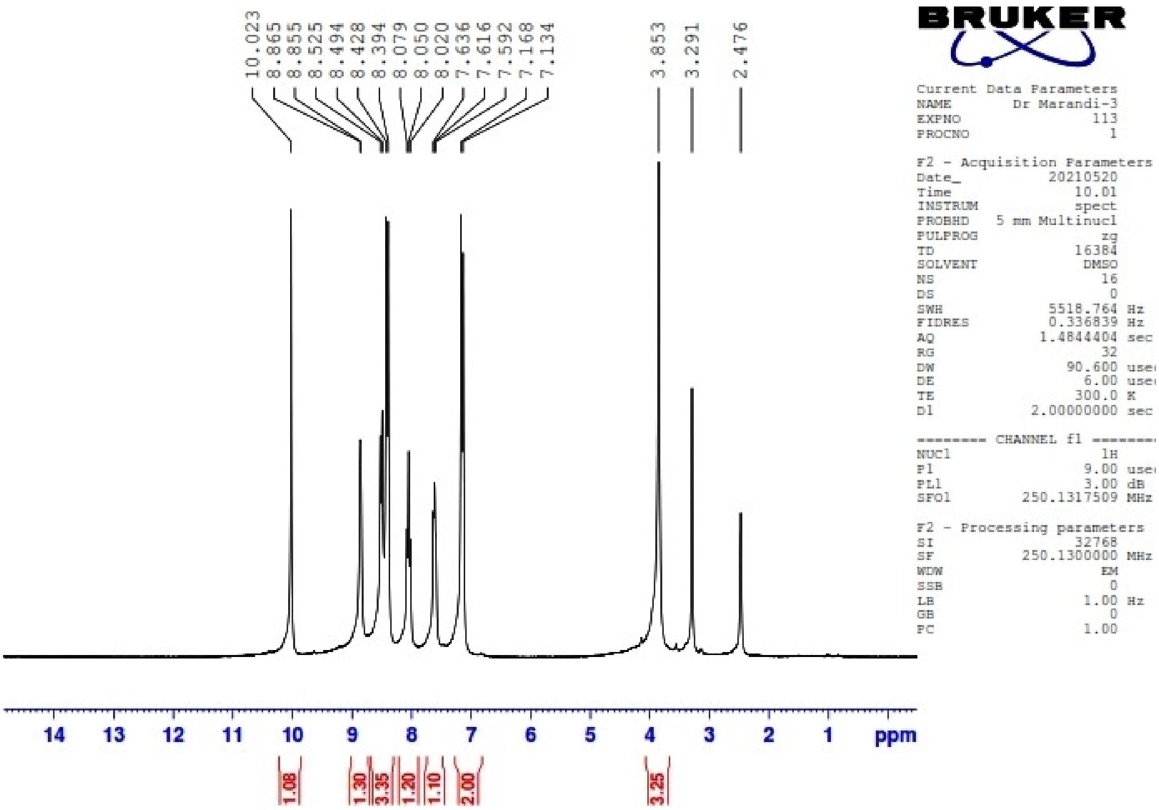

Figure S3: 250 MHz ^{1}H NMR spectrum of **1** in DMSO-d_6.

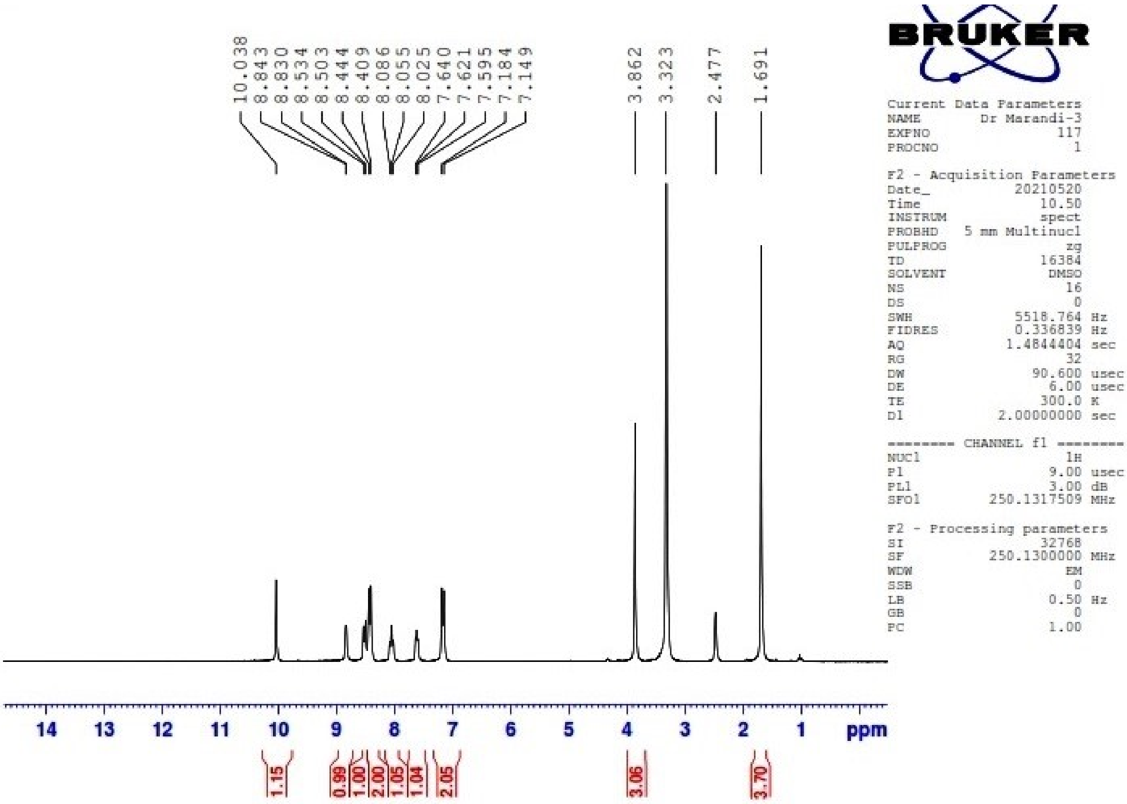

Figure S4: 250 MHz ^{1}H NMR spectrum of **2** in DMSO-d_6.

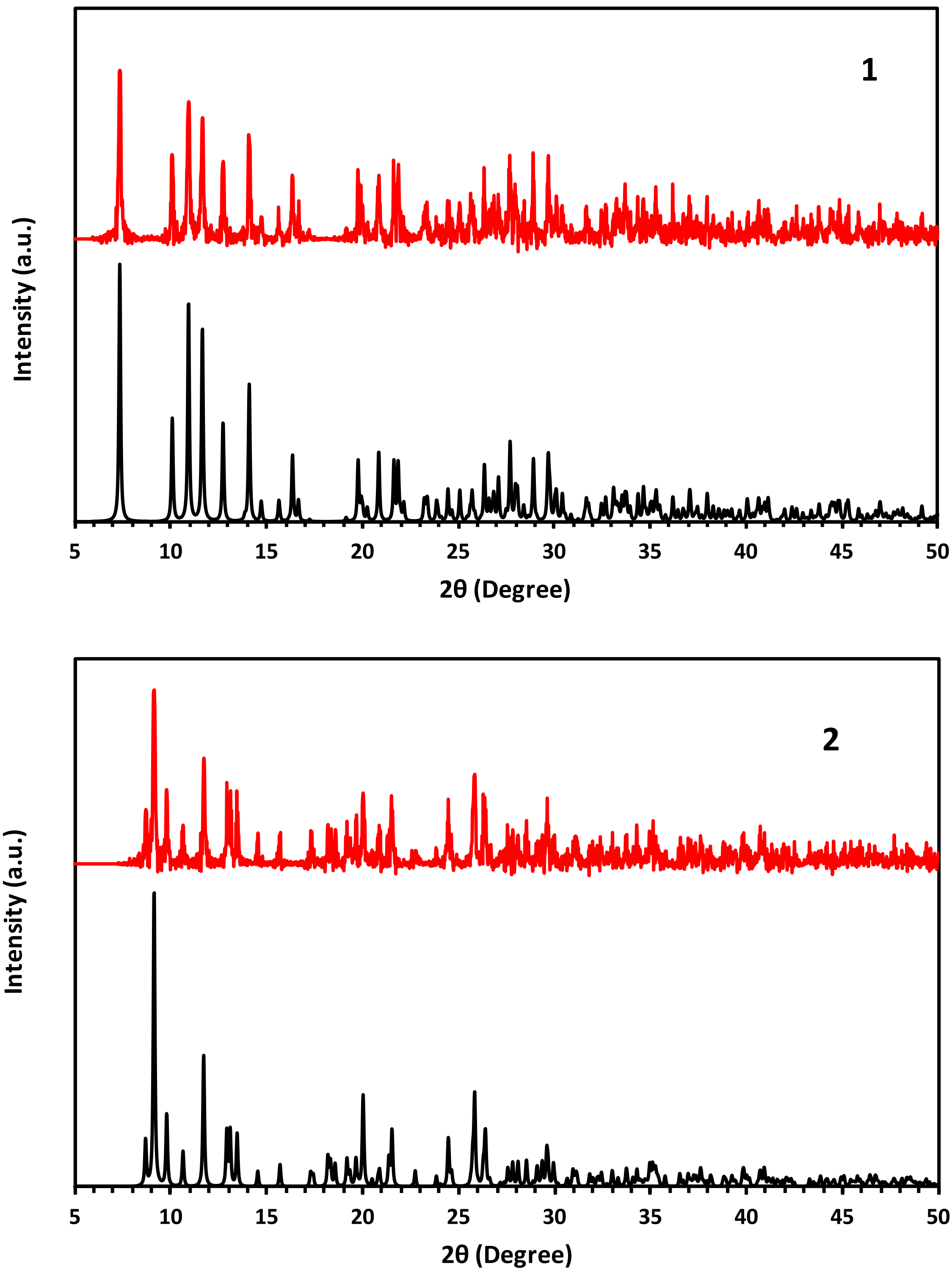

Figure S5. Experimental (in red) and simulated (in black) powder X-ray diffraction patterns of **1** and **2**. The simulated pattern is based on the structure refined by single-crystal X-ray diffraction analysis.

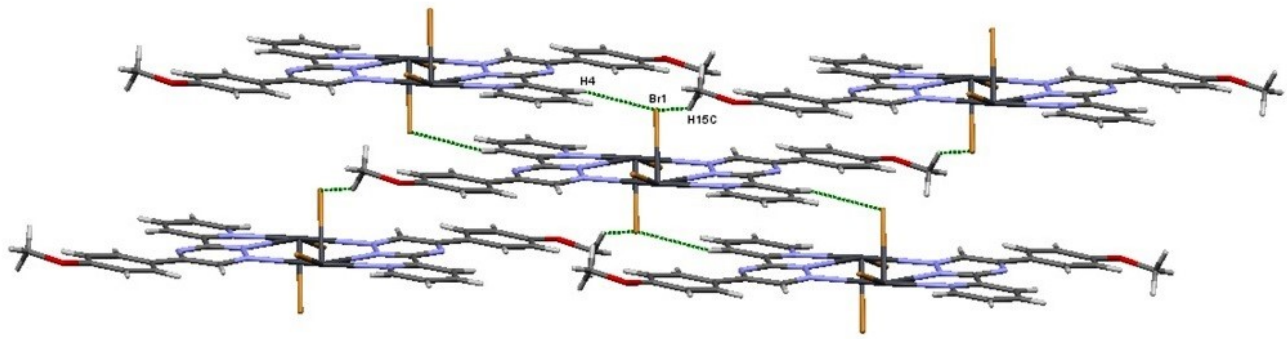

(a)

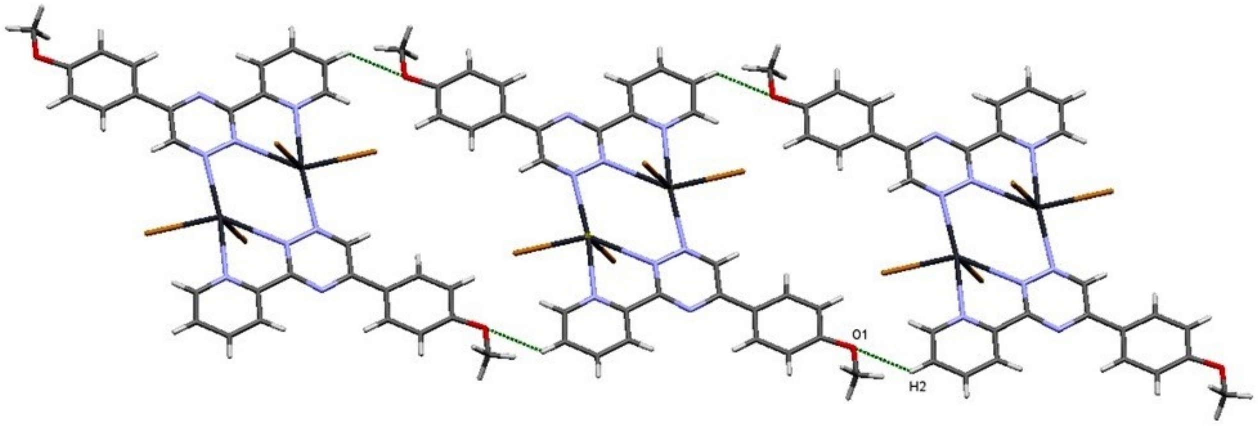

(b)

Figure S6: Crystal packing in **1** by a) C-H···Br b) C-H···O intermolecular interactions.

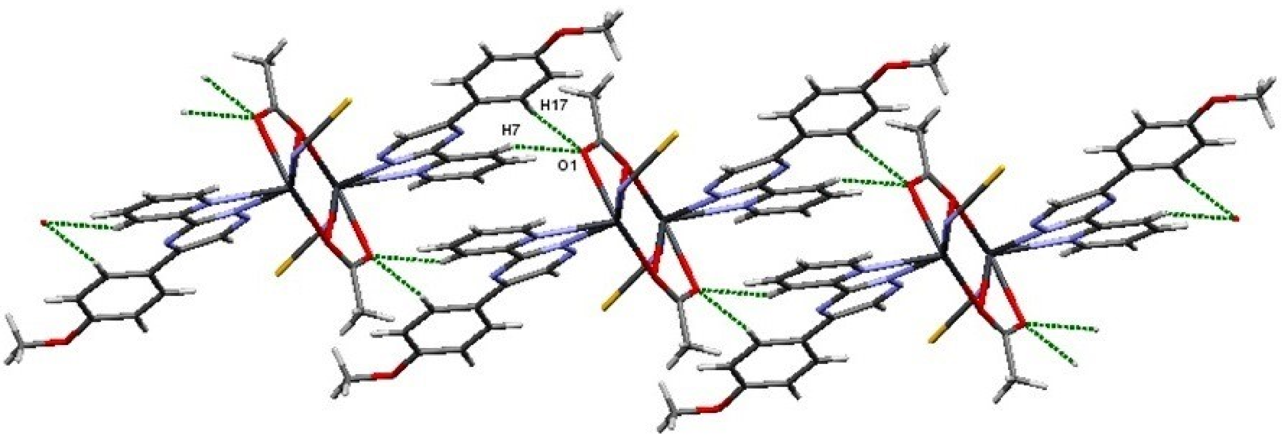

Figure S7: Crystal packing by C-H···O intermolecular interactions and π-π stacking in **2**
